# Supplementary material for: Conditional chemoconnectomics (cCCTomics) as a strategy for efficient and conditional targeting of chemical transmission
Source: eLife. 2024 Apr 30;12:RP91927. doi: 10.7554/eLife.91927 (PMC11060718; doi:10.7554/eLife.91927)
Supplement: Figure 3—source data 3. [file elife-91927-fig3-data3.zip › Figure 3 source data3 part 2.zip!/Figure 3-Source data 3-part2/1002-9-CRISPResso_Report_e53dd8m5.zip!/CRISPResso_on_1002_11/2a.Nucleotide_percentage_quilt.pdf]

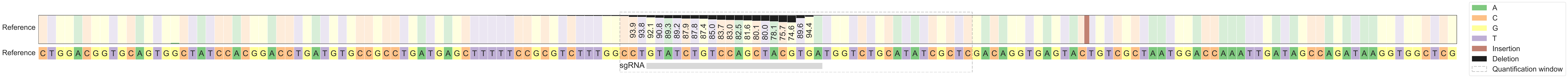
Reference
93.9 93.8 92.1 90.8 89.3 89.2 87.9 87.8 87.4 85.0 83.7 83.0 82.5 81.6 80.1 80.0 78.1 75.7 74.6 89.6 94.4
Reference
CTGGACGGTGCAGTGGCTATCCACGGACCTGATGTGCCGCCTGATGAGCTTTTTCCGCGTCTTTGGCCTGTATCTGTCCAGCTACGTGATGGTCTGCATATCGCTCGACAGGTGAGTACTGTCGCTAATGGACCAAATTGATAGCCAGATAAGGTGGCTCG
sgRNA
A
C
G
T
Insertion
Deletion
Quantification window
